# Comparison of Dose and Effectiveness of a Single-Session Ultrasound-Guided High-Intensity Focused Ultrasound Ablation of Uterine Fibroids With Different Sizes

**DOI:** 10.3389/fonc.2021.725193

**Published:** 2021-12-21

**Authors:** Mei-Jie Yang, Ren-Qiang Yu, Jin-Yun Chen, Zhi-Biao Wang

**Affiliations:** ^1^ State Key Laboratory of Ultrasound in Medicine and Engineering, College of Medical Informatics, Chongqing Medical University, Chongqing, China; ^2^ Department of Radiology, the First Affiliated Hospital of Chongqing Medical University, Chongqing, China; ^3^ State Key Laboratory of Ultrasound in Medicine and Engineering, Chongqing Medical University, Chongqing, China; ^4^ Department of Oncology, the First Affiliated Hospital of Chongqing Medical University, Chongqing, China

**Keywords:** high-intensity focused ultrasound, ultrasound ablation, uterine fibroids, NPVR, EEF

## Abstract

**Purpose:**

This study aimed to compare the dose and effectiveness of ultrasound-guided high-intensity focused ultrasound (USgHIFU) ablation of uterine fibroids with different sizes and explore the effect of uterine fibroid size on dose, which provided dose evaluation for clinicians in accordance with the size of uterine fibroids.

**Materials and Methods:**

A total of 1,000 patients with symptomatic uterine fibroids who received a single-session USgHIFU treatment were enrolled in this study. The size of fibroids was divided into seven groups: 3–4 cm, 4–5 cm, 5–6 cm, 6–7 cm, 7–8 cm, 8–9 cm, and 9–11 cm. The dose was expressed on the basis of the energy efficiency factor (EEF) as the energy required for ablation per unit volume of tissue, and the non-perfused volume ratio (NPVR) was used to assess the effect of HIFU ablation.

**Results:**

The median NPVR of 88.3% (IQR: 80.3%–94.8%) was obtained, and no significant difference was observed among the seven groups. The classification of T2-weighted image signal intensity fibroids in the 4–5 cm group was compared with that in the 6–7 cm and 8–9 cm groups, and the difference was significant (*p* < 0.05). However, the proportion of T2WI hyperintense signal fibroids had no significant difference among the seven groups (*p* > 0.05). The median EEF was 3.88 J/mm^3^, and a significant difference was observed among the seven groups of EEF (*p* < 0.05). The EEF of groups with a fibroid size less than 6 cm was more than double the EEF of groups with a fibroid size above 6 cm. In addition, the EEF of groups with a fibroid size of 4–5 cm and 3–4 cm was 3–4 times higher than those with a fibroid size above 7 cm (*p* < 0.05).

**Conclusions:**

A single-session HIFU ablation for uterine fibroids of 3–11 cm can obtain an NPVR of more than 80%. The EEF decreased with the increase of the size of uterine fibroids. A fibroid size of 6.5 cm was considered as a clinical meaningful point affecting EEF.

## Introduction

Uterine fibroids (UFs) are common benign tumors in women of childbearing age. Many patients complain of clinical symptoms such as menorrhagia, irregular bleeding, pelvic pain, or infertility (1–3), which seriously affect their quality of life. At present, the treatment for UFs includes surgery, medicine, uterine artery embolization, ultrasound-guided high-intensity focused ultrasound (USgHIFU), and magnetic resonance-guided high-intensity focused ultrasound (4–8). The high-intensity focused ultrasound (HIFU) treatment is widely used in clinical practice. This noninvasive technique is safe and effective, and it can preserve organs without damaging endocrine function (9, 10). HIFU beam focuses on the tumor to induce coagulation necrosis, thereby treating UFs (11, 12). The assessment of ultrasound dosimetry and ablation effect is important to ensure the safe and effective treatment of HIFU. The energy efficiency factor (EEF) refers to the energy required for ablation per unit volume of tissue and reflects the regularity of the energy–effect relationship of HIFU ablation (13). The non-perfused volume ratio (NPVR) of HIFU ablation of UFs refers to the ratio of the volume of the non-perfusion area in the postoperative enhanced MR image to the volume of the fibroids. In addition, NPVR is related to symptom relief, which can be used as the imaging gold standard for evaluating the effect of HIFU ablation (14–16).

The clinical study of HIFU ablation of UFs was approved by the Ethics Committee of the First Affiliated Hospital of Chongqing Medical University in 2006. The earliest clinical results were reported in the *Chinese Journal of Obstetrics and Gynaecology* (17). Meanwhile, continuous studies were conducted in dosimetry and efficiency evaluation. From December 2006 to January 2009, Chen JY explored the dose-related factors affecting ultrasound ablation of UFs in 142 patients. From October 2010 to January 2013, Zhao WP studied the feasibility of HIFU ablation of T2-weighted image (T2WI) hyperintense UFs in 491 patients, the biological characteristics of UFs with different T2WI signals, and the prediction of USgHIFU ablation of T2WI hyperintense UFs by dynamic enhanced magnetic resonance imaging (MRI) (14, 18, 19). Yin N conducted a safety study on 861 cases of UFs treated with HIFU from January 2013 to December 2015 and reported the effect of abdominal wall scar on HIFU ablation of UFs (20). Based on our previous research, a total of 1,000 patients with UFs who underwent continuous USgHIFU from January 2013 to June 2018 were included in this study to explore the factors affecting the ablative efficiency of HIFU ablation and analyze the relative importance of these factors to the efficiency; accurate ablative efficiency prediction and the factors affecting ablative efficiency could optimize patient screening for HIFU ablation of UFs (21). Previous studies have focused on assessing the ablation energy and ablation effect based on tissue in the acoustic pathways, MRI signal intensity, structure, blood supply, and function of target tissues (22). Previous studies have shown that the EEF is negatively correlated with the size of UFs, and the NPVR has no relationship with the size of UFs (23–25). However, the difference in dose and effect of USgHIFU ablation of UFs with different sizes remains unclear. The dosage of USgHIFU ablation of UFs with different sizes has not been studied. Therefore, this study compared the dose and effect of a single-session HIFU ablation of UFs with different sizes and provided a basis for preoperative screening of patients. Furthermore, this study explored the effect of UF size on dose, which provided dose evaluation for clinicians in accordance with the size of UFs.

## Materials and Methods

### Patients

The study protocol was approved by the Ethics Committee of the First Affiliated Hospital of Chongqing Medical University. Patients with UFs who received single-session USgHIFU at the First Affiliated Hospital of Chongqing Medical University from January 2013 to June 2018 were enrolled in this study (IRB number: Ethics 16, 2006; approval date: August 18, 2006). Prior to enrollment, the fibroid status and treatment plan were evaluated by a gynecologist, a radiologist, and a HIFU physician. Before HIFU treatment, the details of the treatment were discussed with all patients who signed a consent form. This retrospective study was approved by our institutional review board, and informed consent was waived because the data were anonymized. All procedures used in this study were in accordance with the ethical standards and Declaration of Helsinki.

The inclusion criteria were as follows: (1) premenopausal patients over 18 years old; (2) patients could communicate with the medical staff during procedure; (3) patients agreed to undergo pre-treatment and post-treatment enhanced MRI scanning; and (4) the size of the UFs was between 3 and 11 cm. In this study, if the patient had multiple fibroids, then only the largest fibroid was selected for investigation.

The exclusion criteria were as follows: (1) patients who were contraindicated for MRI scanning or gadolinium-injection solution; (2) patients with significant degenerative fibroids or suspected uterine malignancy assessed by enhanced MRI; (3) patients with special category of fibroids, such as pedunculated subserous or submucosal fibroids; (4) patients with scar tissue in the acoustic pathway, causing evident attenuation of the B-mode ultrasound after the detection of tissues (sound attenuation width ≥15 mm); and (5) patients who cannot lie in a prone position for 2 h.

### MRI Evaluation

All patients received MRI scans before and within 1 week after the treatment. A series of T1WI, T2WI, and enhanced T1WI were performed using the 3.0-T MRI system (GE Medical system, Milwaukee, WI, USA).

The UFs and uterus were measured on T2WI to obtain data from three dimensions: longitudinal diameter (D1) and anteroposterior diameter (D2) were measured in sagittal T2WI, whereas transverse diameter (D3) was measured in axial T2WI. The size of the fibroid was the largest in D1, D2, and D3. The non-perfused volume (NPV) was considered as the fibroid necrosis volume, which was evaluated on post-treatment enhanced MRI (15). The post-treatment fibroid volume was measured on T2WI. The fibroid volume, uterus volume, and NPV were calculated using the following equation: V = 0.5233 × D1 × D2 × D3 (26). The NPVR was calculated as follows: NPV/post-treatment fibroid volume × 100%. Funaki types were clinically used as categories to classify the intensity of UFs based on T2-weighted MR images: hypointense, isointense, and hyperintense (27). Funaki III represents hyperintense signal fibroids, which signal intensity equal to or higher than that of myometrium. Based on the degree of enhancement of UFs compared with that of the myometrium 60 s after gadolinium injection (28, 29), the enhancement of T1-weighted images (T1WI) was divided into three types: slight enhancement, regular enhancement, and irregular enhancement. All MRI examination data were evaluated by three experienced radiologists.

### Ultrasound-Guided HIFU Ablation

HIFU ablation was performed by HIFU licensed physicians with at least 3 years of HIFU clinical experience using the model-JC Focused Ultrasound Tumor Therapeutic System (Chongqing Haifu Medical Technology Co., Ltd., Chongqing, China). The equipment was combined with an ultrasonic imaging device, which provided real-time guiding during ablation. The experimental parameters used in this study were as follows: the operating frequency of the US transducer was 0.8 MHz, and energy was adjustable in the range of 200–400 W. Circulating degassed water was used as the coupling medium, and the focal region was 1.5 mm × 1.5 mm × 10.0 mm. The patients lay in a prone position on the HIFU therapy table, with the anterior abdominal wall in contact with degassed water. A catheter was inserted into the bladder and degassed normal saline was filled to control the volume of bladder. A degassed water balloon was placed between the abdominal wall and transducer to compress and push away the bowel from the acoustic pathway during treatment.

**Figure 1:**
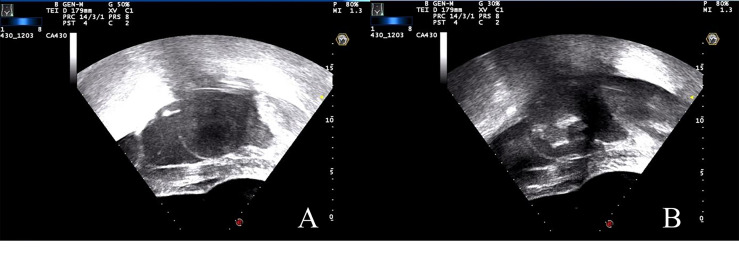
US images during the USgHIFU procedure. **(A)** Ultrasound image showed a uterine fibroid with hypoecho before treatment. **(B)** A gray scale change was observed during sonication.

Fentanyl-midazolam was used to keep conscious sedation. The ablation results were monitored on the basis of the gray changes in the target area displayed by ultrasound imaging, and sonication was terminated when the increased gray scale covered the planned ablation area (18, 30). For patients with multiple fibroids, the main fibroid was treated initially, and other fibroids were ablated within 3 h. Pre-procedure, intra-procedure, and post-procedure ultrasound images are shown in **Figure 1**. All the complications were recorded and graded in accordance with the SIR classification standard by the Society of Interventional Radiology. In SIR classification, grades C–F were considered as major complications (10, 31).

### Dosimetric Analysis

The dosimetric indicators were as follows: ultrasonic power (W, average power of the ultrasonic transducer), treatment time (min, the time from the first sonication to the last sonication), sonication power (W, average acoustic power during ablation), and sonication time (seconds, total time of sonication).

Dosage was expressed as EEF: EEF = *η* × P × t/V (J/mm^3^), where *η* represents the focus coefficient (= 0.7); P represents the sonication power; t represents the sonication time, and V indicates the NPV (24).

### Statistical Analysis

The data that followed normal distribution were presented as mean with SD; otherwise, they were presented as median with 25th and 75th interquartile ranges (IQR). The categorical variables were described by the number and proportion of individuals. The Kolmogorov–Smirnov and Levene tests were used to test whether the data were normally distributed and homogenous of variance. The Spearman test was used to evaluate the correlation between the UF size and EEF. The data following non-normal distribution were applied to non-parametric tests (Kruskal–Wallis and Wilcoxon tests). Piecewise regression was used to examine the impact of UF size on EEF. Stepwise regression was used to construct the regression equation between EEF and UF size in different segments. The level of significance applied for all tests was set at *p* < 0.05. Statistical analyses were performed by R, version 3.6 (R Core Team, Vienna, Austria).

## Results

### Baseline Characteristics of Patients

A total of 1,000 patients with fibroids were enrolled for analysis, with a median age of 40 years (IQR: 35–44 years). Surgical scars of the lower abdomen were observed in 241 patients, including scars from previously open myomectomy and cesarean section. The size of the UFs was 5.7 cm (IQR: 4.8–6.9 cm), and the volume of the uterus was 245.8 cm^3^ (IQR: 175.1–339.1 cm^3^) (**Table 1**).

**Table 1:** Baseline characteristics of patients.

Variable	Data
Age (years)*	40 (35–44)
Height (cm)*	158 (155–160)
BMI (kg/m^2^)*	22.2 (20.7–24)
Thickness of rectus abdominis (mm)*	9 (7.5–11)
Thickness of subcutaneous fat layer (mm)*	16.1 (12.3–21.2)
Uterine volume(cm^3^)*	245.8 (175.1–339.1)
Location of uterus(anteverted/median/retroverted) (*n*)	635/97/268
Largest diameter of uterine fibroids (cm)*	5.7 (4.8–6.9)
Type of uterine fibroids (submucous/subserous/intramural) (*n*)	165/185/650
Location of uterine fibroids (anterior/posterior/lateral/fundus) (*n*)	400/263/275/62
Signal intensity on T2WI (hypointense/isointense/hyperintense) (*n*)	322/398/280
Distance from center of fibroid to sacrum (mm)*	47.6 (38.5–61.05)
Distance from ventral side of fibroid to skin (mm)*	39.7 (28.5–55)

*Data are median (interquartile range); BMI, body mass index.

### Results of Ultrasound Ablation

**Figure 2:**
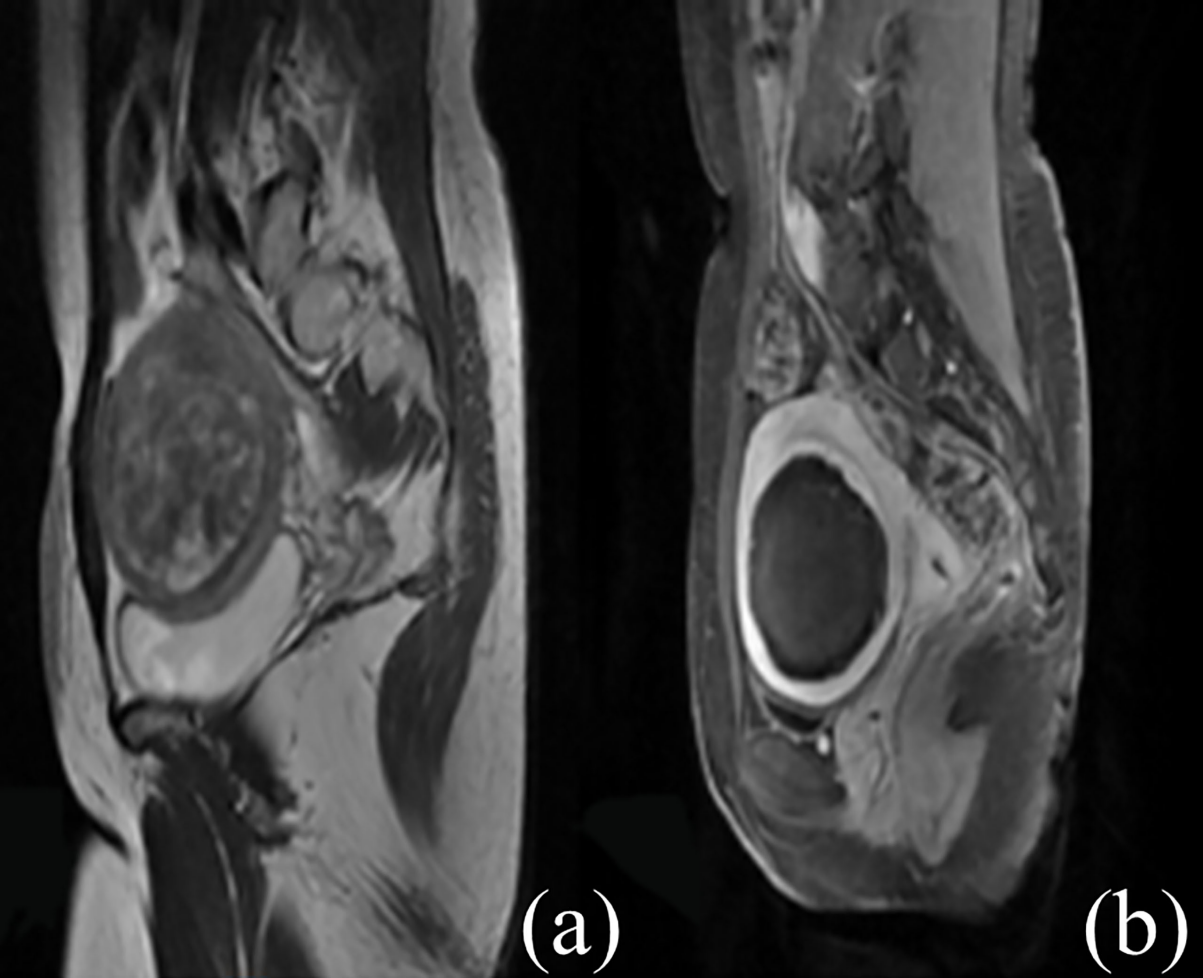
Contrast-enhanced MR images before and after HIFU treatment. **(A)** The fibroid was subserous, anterior, and hyperintense before treatment. **(B)** The non-perfused volume was shown inside uterine fibroid after treatment.

All the patients completed ultrasound ablation. The pre-procedure and post-procedure MR images within 7 days are shown in **Figure 2**. The median ultrasonic power was 400 W; the median sonication time was 852 s (IQR: 544–1,348 s); the median total dosage was 328,440.0 J (IQR: 206,772.5–528,800.0 J); the median NPV ratio was 88.3% (IQR: 80.3%–94.8%), and the median EEF was 3.88 J/mm^3^ (IQR: 2.24–6.32 J/mm^3^) (**Table 2**).

**Table 2:** The parameters and results of ultrasound ablation.

Parameter	Data*
Ultrasonic power (W)	400 (395–400)
Sonication time (s)	852 (544–1,348)
Total dose (J)	328,440.0 (206,772.5–528,800.0)
NPV (cm^3^)	75.8 (36.2–96.2)
NPVR (%)	88.3 (80.3–94.8)
EEF (J/mm^3^)	3.88 (2.24–6.32)

NPV, non-perfused volume; NPVR, non-perfused volume ratio; EEF, energy efficiency factor.

*Data are median (interquartile range).

### Comparison of NPVR and Baseline Characteristics Among UFs of Different Sizes

The size of UFs was divided into seven groups: 3–4 cm, 4–5 cm, 5–6 cm, 6–7 cm, 7–8 cm, 8–9 cm, and 9–11 cm. The patients in the 3–4 cm group were younger than those in the 4–5 cm, 6–7 cm, and 8–9 cm groups (*p* < 0.05). The classification of T2WI signal intensity fibroids (Funaki I, II, and III) in the 4–5 cm group was compared with that in the 6–7 cm and 8–9 cm groups, and the difference was significant (*p* < 0.05). However, the proportion of T2WI hyperintense signal fibroids (Funaki III) had no significant difference among the seven groups (*p* > 0.05). The enhancement type of T1WI fibroids in the 4–5 cm group was compared with that in the 6–7 cm, 7–8 cm, and 8–9 cm groups, and the difference was significant (*p* < 0.05). No statistically significant difference in BMI and NPVR was observed among the seven groups (*p* > 0.05, **Table 3**).

**Table 3:** Comparison of NPVR and baseline characteristics among fibroids of different sizes.

Groups	3–4 cm	4–5 cm	5–6 cm	6–7 cm	7–8 cm	8–9 cm	9–11 cm	*p*-value
Number of cases (%)	58 (5.8)	221 (22.1)	262 (26.2)	214 (21.4)	140 (14.0)	70 (7.0)	35 (3.5)	
Age	40 (34–44)	42 (37–45)	40 (34–44)	42 (37–44)	41 (37–45)	42 (37–46)	41 (38–44)	0.000***
BMI	21.6 (20.0–22.8)	22.1 (20.7–24.1)	22.4 (20.6–24.2)	22.3 (20.5–24.2)	21.8 (20.6–23.6)	22.3 (21.1–24.6)	23.0 (21.4–24.4)	0.241
Signal intensity on T2WI (*n*, %)								<0.000***
Hypointense	22 (37.9)	91 (41.1)	91 (34.7)	56 (26.4)	41 (29.3)	13 (18.6)	8 (22.9)	
Isointense	26 (44.8)	80 (36.5)	101 (38.6)	84 (39.6)	58 (41.4)	32 (45.7)	15 (42.8)	
Hyperintense	10 (17.3)	50 (22.6)	70 (26.7)	74 (34.6)	41 (29.3)	25 (35.7)	12 (34.3)	
Enhancement type on T1WI (*n*, %)								0.048*
Slight	29 (53.7)	120 (52.6)	170 (60.9)	132 (63.5)	82 (62.6)	39 (63.9)	24 (61.5)	
Irregular	7 (13.0)	33 (14.5)	40 (14.3)	35 (16.8)	24 (18.3)	8 (13.1)	5 (12.8)	
Regular	18 (33.3)	75 (32.9)	69 (24.8)	41 (19.7)	25 (19.1)	14 (23.0)	10 (25.7)	
NPVR(%)	89.4 (80.0–94.8)	88.0 (80.2–95.3)	88.3 (80.9–95.2)	88.6 (80.0–94.3)	88.9 (82.4–95.1)	84.9 (74.1–91.7)	90.6 (81.6–97.5)	0.102

*Data are median (interquartile range); BMI, body mass index; NPVR, non-perfused volume ratio. ***p<0.000; *p<0.05.

Age: Group 3–4 cm vs. Group 6–7 cm: p = 0.005; Group 3–4 cm vs. Group 7–8 cm: p = 0.036; Group 3–4 cm vs. Group 8–9 cm: p = 0.011.

T2WI: Group 4–5 cm vs. Group 6–7 cm: p = 0.014; Group 4–5 cm vs. Group 8–9 cm: p = 0.018.

T1WI: Group 4–5 cm vs. Group 6–7 cm: p = 0.039; Group 4–5 cm vs. Group 7–8 cm: p = 0.006; Group 4–5 cm vs. Group 8–9 cm: p = 0.019.

The statistical test of the overall data was conducted by Kruskal–Wallis test. Statistical tests of data between groups were conducted by Wilcoxon test.

### Comparison of Dose Among UFs of Different Sizes

**Figure 3:**
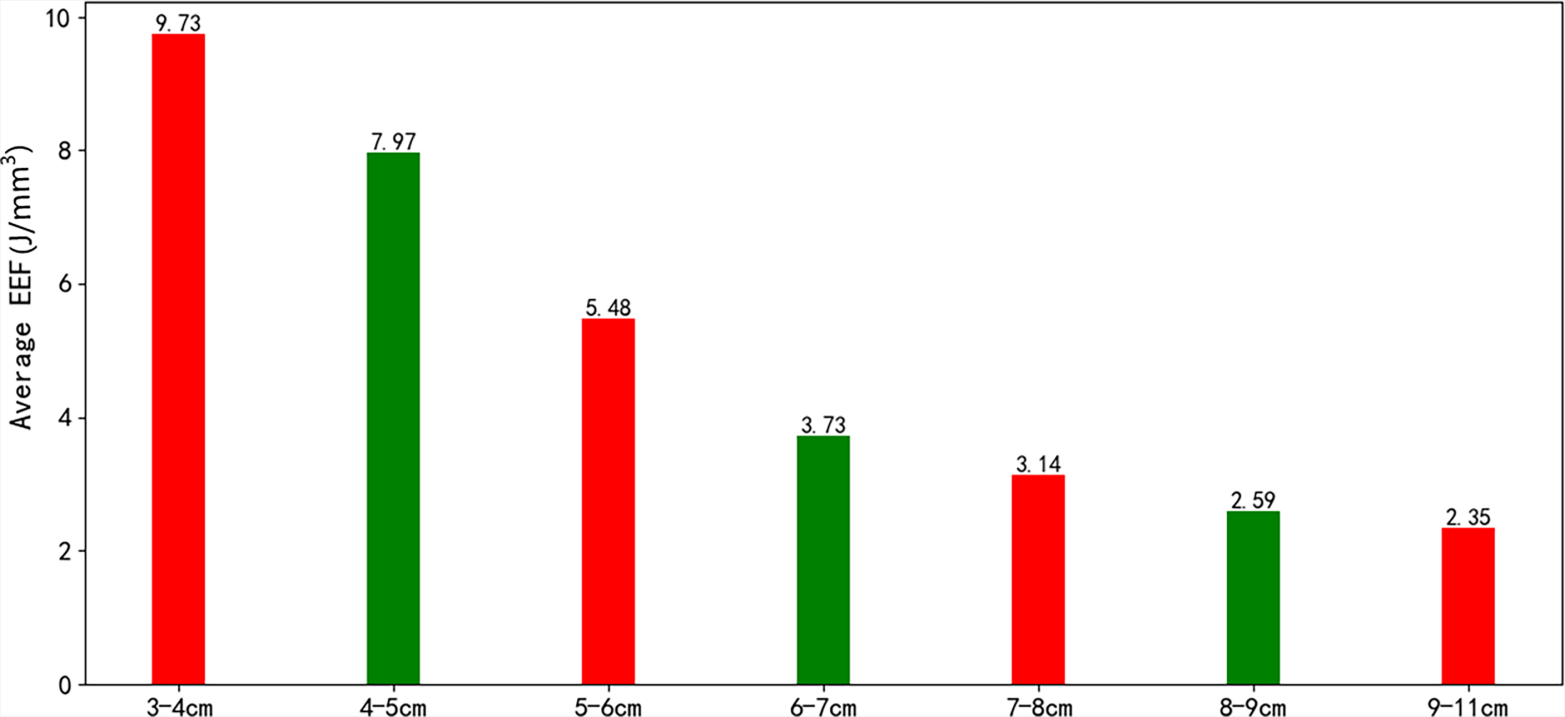
The average EEF among fibroids of different sizes.

**Figure 4:**
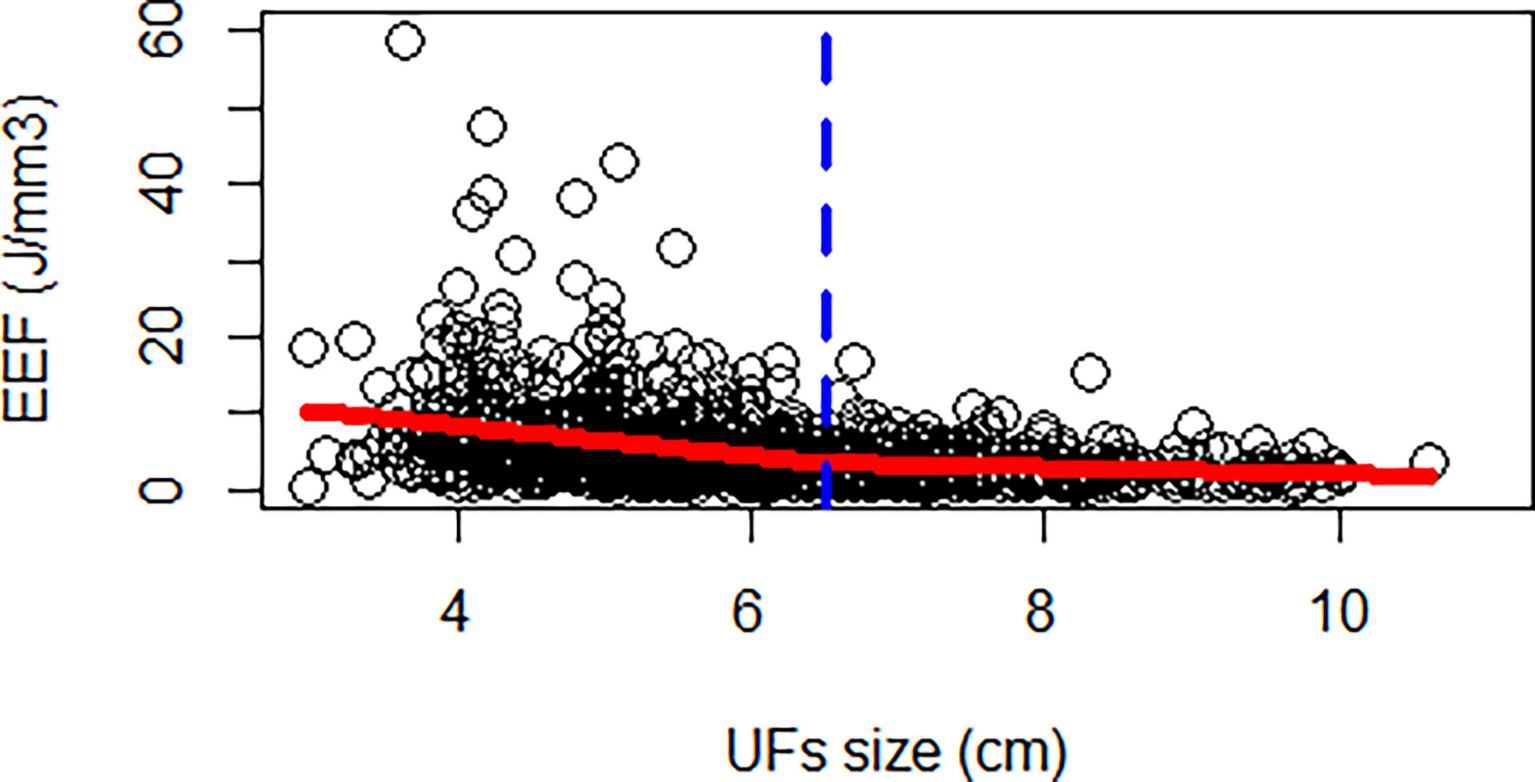
A piecewise regression of UF size.

The correlation coefficient between the EEF and fibroid size was −0.44 (*p* < 0.000). As the size of UFs increased from 3–4 cm to 9–11 cm, the corresponding average EEF (J/mm^3^) was 9.73, 7.97, 5.47, 3.73, 3.14, 2.59, and 2.35 J/mm^3^. Significant differences in EEF were observed among the seven groups (*p* < 0.05). The EEF of the 3–4 cm group was significantly higher than that in the 5–6 cm, 6–7 cm, 7–8 cm, 8–9 cm, and 9–11 cm groups (*p* < 0.05). The EEF of the 4–5 cm group was significantly higher than that in the 5–6 cm, 6–7 cm, 7–8 cm, 8–9 cm, and 9–11 cm groups (*p* < 0.05). The EEF of the 5–6 cm group was significantly higher than that in the 6–7 cm, 7–8 cm, 8–9 cm, and 9–11 cm groups (*p* < 0.05). The EEF of groups with a fibroid size less than 6 cm was more than double the EEF of groups with larger fibroid size. In addition, the EEF of the 4–5 cm and 3–4 cm groups was 3–4 times higher than those with a fibroid size above 7 cm (*p* < 0.05, **Table 4**). The EEF was lower with the increase of UFs in significantly different groups (**Figure 3**). Therefore, a UF size of 6.5 cm was considered as a clinical meaningful point based on the segmented package of R language (**Figure 4**). The piecewise regression equations were constructed using stepwise regression: when UF size < 6.5 cm, EEF = 12.71 − 2.08X_UFs_ + 1.45 × X_T2WI_ + 0.73 × X_T1WI_; otherwise, EEF = 4.72 − 0.44 × X_UFs_ + 0.40 × X_T2WI_ + 0.47 × X_T1WI_ (**Table 5**).

**Table 4:** Comparison of dose among fibroids of different sizes (J/mm^3^).

Groups	3–4 cm	4–5 cm	5–6 cm	6–7 cm	7–8 cm	8–9 cm	9–11 cm
Mean ± SD	9.73 ± 8.51	7.97 ± 6.86	5.47 ± 4.62	3.73 ± 2.54	3.14 ± 2.15	2.59 ± 2.18	2.35 ± 1.53
Median	7.09	5.96	4.36	3.28	2.50	2.03	2.13
First quartile (Q1)	5.11	3.61	2.65	1.84	1.50	1.36	1.03
Third quartile (Q3)	10.69	9.70	6.71	4.68	4.30	3.41	2.90
Minimum	1.51	0.70	0.49	0.40	0.23	0.59	0.46
Maximum	58.58	47.78	42.95	16.80	10.90	15.13	6.04

SD, Standard deviation; First quartile (Q1): The number 25% after the smallest to the largest of all values in the sample; Third quartile (Q3): The number 75% after the smallest to the largest of all values in the sample.

The statistical test of the overall data was conducted by Kruskal–Wallis test (p < 0.000).

Statistical tests of data between groups were conducted by Wilcoxon test.

Group 3–4 cm vs. Group 5–6 cm: p < 0.000; Group 3–4 cm vs. Group 6–7 cm: p < 0.000; Group 3–4 cm vs. Group 7–8 cm: p < 0.000; Group 3–4 cm vs. Group 8–9 cm: p < 0.000; Group 3–4 cm vs. Group 9–11 cm: p < 0.000.

Group 4–5 cm vs. Group 5–6 cm: p < 0.000; Group 4–5 cm vs. Group 6–7 cm: p < 0.000; Group 4–5 cm vs. Group 7–8 cm: p < 0.000; Group 4–5 cm vs. Group 8–9 cm: p < 0.000; Group 4–5 cm vs. Group 9–11 cm: p < 0.000.

Group 5–6 cm vs. Group 6–7 cm: p < 0.000; Group 5–6 cm vs. Group 7–8 cm: p < 0.000; Group 5–6 cm vs. Group 8–9 cm: p < 0.000; Group 5–6 cm vs. Group 9–11 cm: p < 0.000.

Group 6–7 cm vs. Group 8–9 cm: p < 0.000; Group 6–7 cm vs. Group 9–11 cm: p = 0.003.

**Table 5:** Piecewise regression model through stepwise regression.

	Estimate	Std. Error	*t* value	*p*
UFs size < 6.5 cm				
Intercept	12.72	1.48	8.58	<0.000***
UFs size	−2.08	0.27	−7.70	<0.000***
Signal intensity on T2WI	1.45	0.20	7.41	<0.000***
Enhancement type on T1WI	0.73	0.25	2.96	0.003**
UFs size ≥ 6.5 cm				
Intercept	4.72	1.06	4.47	<0.000***
UFs size	−0.44	0.13	−3.28	0.001**
Signal intensity on T2WI	0.40	0.11	3.62	0.000***
Enhancement type on T1WI	0.47	0.15	3.09	0.002**

***p<0.000; **p<0.001.

### Complications

No major complications defined as SIR classifications C–F were found.

## Discussion

Focused ultrasound ablation surgery of UFs may provide effective management until menopause because of its rapid recovery and low risks of complications (32). The NPVR, as a measure of technical success, is recognized as a predictor of clinical outcome for HIFU ablation of fibroids. The re-intervention rates closely correlated with the mean NPVR achieved (33, 34). A NPVR of at least 90% or even almost 100% of the fibroid tumor volume was recommended without compromising safety (35, 36). However, given the histological characteristics of UFs and technical limitations, not all UFs could obtain an NPVR of 90% to 100% (35). Park MJ reported that during USgHIFU ablation of UFs, achieving an immediate NPVR of at least 80% is safe, with greater tumor volume shrinkage compared with cases with a low NPVR (36). A number of studies have shown that the average or median NPVR of USgHIFU ablation of UFs has reached more than 80% (7, 25, 37). Evaluating the tissue characteristics of the UFs and technical limitations in the screening phase might reduce the risk of an unsuccessful HIFU treatment outcome of UFs (22).

HIFU is an alternative treatment option for patients with multiple UFs and large fibroids who want to preserve their uterus and fertility (38). The size of UFs can reflect the histological characteristics of UFs. With the increase of UFs, the blood supply becomes relatively insufficient, and degeneration occurs. Given the lack of measurement of the thermal dose, studying the dose–effect relationship using the thermal dose during HIFU will be impossible. This study used the EEF as a quantitative indicator to mark the dose for the HIFU ablation of UFs. Previous studies have found that the dose of ultrasound ablation is negatively correlated with the size of fibroids (24, 25). Therefore, the dose and effect of ultrasound ablation of UFs with different sizes were studied, which will be of great help in selecting appropriate patients for HIFU ablation. Piecewise regression was used to explore important meaningful points of UF size, which could provide a basis for clinicians to use doses in accordance with the UF size.

In this study, of the 1,000 patients who had either single fibroids or multiple fibroids, only the largest fibroid per patient was included for analysis. The median NPVR of 88.3% (IQR: 80.3%–94.8%) was obtained with no major complication. The potential complications of HIFU treatment include peripheral tissue injury and systemic HIFU-related adverse events (6, 30, 39). In this study, when high NPVR was obtained, no major complications such as intestinal perforation and nerve injury were observed, which indicated the progress and maturity of clinical techniques and protocols.

The results showed that the proportion of T2WI hyperintense signal fibroids (Funaki III) had no significant difference among the seven groups (*p* > 0.05). In addition, the NPVR had no statistical difference among the seven groups (*p* > 0.05). The results indicated that fibroids of different sizes could obtain satisfactory ablation outcomes with median NPVR ≥ 80%, which were not related to the proportion of fibroids with a T2WI hyperintense signal. These results were inconsistent with those presented in previous studies (23, 35, 40, 41). This difference may be due to the relatively large number of cases in this study, and the clinicians are skilled in HIFU with at least 3 years of experience in our center. Therefore, with the optimization of techniques, fibroids with T2WI hyperintense signal will no longer be used as an indicator for ultrasound ablation.

The EEF indicates the energy required for ablation per unit volume of tissue, and it decreases with the increase of UF size in significantly different groups. Previous studies found that the EEF was negatively correlated with the size of UFs (23, 24).

This study initially analyzed the EEF of seven groups with different fibroid sizes based on relatively large samples and found a significant difference between the EEF of groups with a fibroid size less than 6 cm and that of groups with a fibroid size more than 6 cm. Based on piecewise regression, UF size of 6.5 cm was considered as a clinical meaningful point affecting the EEF. First, UFs had an expansive growth pattern of benign tumors, and with the growth of fibroids, particularly above 7 cm, the internal echo of fibroids became heterogeneous during ultrasound scanning. In addition, the inhomogeneous acoustic tissue properties would increase the scattering and absorption of the acoustic wave. Second, when the diameter of the fibroids increased above 5 cm, the blood supply inside the fibroids was relatively insufficient, which was the main reason for the degeneration of fibroids. However, enhanced MRI did not show microvascular perfusion because of the presence of volumetric effects, and ultrasonic energy was easily deposited in tissues with relatively insufficient blood supply and degeneration. Third, the vascular effect of ultrasound caused vascular necrosis and blood flow blockage, which led to a reduction in ultrasound dose for subsequent tissue ablation. For larger tumors, the thermal injury spread locally to untreated areas, which resulted in NPV being larger than the planned treatment area (42–44). Therefore, for larger fibroids, a relatively low ultrasound dose could achieve a better ablation effect. Previous studies have shown that fibroid with ablated T2WI hyperintense signal requires higher energy (18).

In this study, the EEF of the 8–9 cm group, which had the highest proportion of T2WI hyperintense signal fibroids, remained consistent with the outcomes of the grouping of fibroid sizes. This result may be related to the large sample size of this study, indicating that with the increase of sample size, the effect of T2WI signal of fibroids on dose is not significant.

This study also has some limitations. First, all the cases received HIFU treatment in a single center. Second, HIFU ablation was performed by HIFU licensed physicians with many years of HIFU clinical experience. Some of the doctors had more than 10 years of HIFU clinical experience. Therefore, further multi-center studies are needed to illustrate whether the same results can be obtained in a wide range of clinical applications. At present, the factors affecting dose and efficiency are selected from clinical practice, and the indicators of symptoms and laboratory data for accurate diagnosis of UFs will be further studied.

## Conclusion

A single-session HIFU ablation of UFs of 3–11 cm can obtain more than 80% NPVR with no significant differences among UFs with different sizes. The dose for ablation per unit volume of tissue was decreased with the increase of UF size, and it was not affected by the different proportion of T2WI hyperintense signal fibroids. The EEF of a single-session HIFU ablation of UFs with a fibroid size of 3–6 cm was 2–4 times higher than those with a fibroid size above 7 cm. Therefore, a UF size of 6.5 cm was considered as the clinical meaningful point affecting EEF.

## Data Availability Statement

The raw data supporting the conclusions of this article will be made available by the authors, without undue reservation.

## Ethics Statement

The studies involving human participants were reviewed and approved by the Ethics Committee of the First Affiliated Hospital of Chongqing Medical University. The patients/participants provided their written informed consent to participate in this study.

## Author Contributions

M-JY contributed to the drafting of the article or critical revision for important intellectual content and the data analysis, statistical analysis, manuscript preparation, manuscript editing, and manuscript review. R-QY contributed to the data interpretation of image. J-YC agreed to be accountable for all aspects of the work in ensuring that questions related to the accuracy or integrity of any part of the article are appropriately investigated and resolved, and approved the version to be published. Z-BW contributed to the conception and design of the data. All authors contributed to the article and approved the submitted version.

## Funding

This study was supported by the Chongqing Medical University special cultivation project on philosophy and social science (grant number X9612) and the Natural Science Foundation of Chongqing (grant number cstc2021jcyj-msxmX0514).

## Conflict of Interest

The authors declare that the research was conducted in the absence of any commercial or financial relationships that could be construed as a potential conflict of interest.

## Publisher’s Note

All claims expressed in this article are solely those of the authors and do not necessarily represent those of their affiliated organizations, or those of the publisher, the editors and the reviewers. Any product that may be evaluated in this article, or claim that may be made by its manufacturer, is not guaranteed or endorsed by the publisher.
